# National substance use patterns on Twitter

**DOI:** 10.1371/journal.pone.0187691

**Published:** 2017-11-06

**Authors:** Hsien-Wen Meng, Suraj Kath, Dapeng Li, Quynh C. Nguyen

**Affiliations:** 1 Department of Health, Kinesiology, and Recreation, College of Health, University of Utah, Salt Lake City, Utah, United States of America; 2 School of Computing, University of Utah, Salt Lake City, Utah, United States of America; 3 Department of Geography, South Dakota State University, Brookings, South Dakota, United States of America; 4 Department of Epidemiology and Biostatistics, University of Maryland School of Public Health, College Park, Maryland, United States of America; Brigham Young University, UNITED STATES

## Abstract

**Purpose:**

We examined openly shared substance-related tweets to estimate prevalent sentiment around substance use and identify popular substance use activities. Additionally, we investigated associations between substance-related tweets and business characteristics and demographics at the zip code level.

**Methods:**

A total of 79,848,992 tweets were collected from 48 states in the continental United States from April 2015-March 2016 through the Twitter API, of which 688,757 were identified as being related to substance use. We implemented a machine learning algorithm (maximum entropy text classifier) to estimate sentiment score for each tweet. Zip code level summaries of substance use tweets were created and merged with the 2013 Zip Code Business Patterns and 2010 US Census Data.

**Results:**

Quality control analyses with a random subset of tweets yielded excellent agreement rates between computer generated and manually generated labels: 97%, 88%, 86%, 75% for underage engagement in substance use, alcohol, drug, and smoking tweets, respectively. Overall, 34.1% of all substance-related tweets were classified as happy. Alcohol was the most frequently tweeted substance, followed by marijuana. Regression results suggested more convenience stores in a zip code were associated with higher percentages of tweets about alcohol. Larger zip code population size and higher percentages of African Americans and Hispanics were associated with fewer tweets about substance use and underage engagement. Zip code economic disadvantage was associated with fewer alcohol tweets but more drug tweets.

**Conclusions:**

The patterns in substance use mentions on Twitter differ by zip code economic and demographic characteristics. Online discussions have great potential to glorify and normalize risky behaviors. Health promotion and underage substance prevention efforts may include interactive social media campaigns to counter the social modeling of risky behaviors.

## Introduction

Use of social networking sites such as Twitter is widespread among people of all ages, though it is particularly popular among younger crowds. Adolescents and young adults are increasingly sharing ideas, searching for opinions, and interacting with their peers through social media. The internet has become a boundless platform for expressing ideas and observing social norms. Through the adoption of smartphones, the diffusion of content sharing and behavior sharing are just several clicks away. A comprehensive report from Pew Research Center indicates 90% of young adults are active users of social media [1].

Researchers are increasingly utilizing social networking sites to develop techniques to track and monitor social interactions [2–4], infectious disease outbreaks [5–7] and public sentiment on various topics [8–12]. Recent studies have incorporated geotagged Twitter data to monitor engagements with substance use. For instance, a recent study found approximately 1 in every 2000 tweets sent was about marijuana [8]. In another study that collected over 7000 smoking-related tweets over a 15-day period, the overall sentiment was positive rather than negative or neutral [12]. A study with hookah-related tweets found 87% of these tweets either normalized or promoted the activity of hookah smoking [9]. These examples suggest social media platforms have great potential to capture public sentiment and track substance activities.

Alcohol consumption is a popular social activity among today’s young crowds. Binge drinking (i.e., consumption of 5 or more drinks in a row) is most popular among young adults between ages 18 and 25 [13]. A recent study that collected nearly 12 million alcohol-related tweets found that the number of pro-drinking tweets exceeded anti-drinking tweets by 10 times [14]. While an alcohol-related tweet does not necessarily indicate actual alcohol consumption, greater alcohol use is associated with more alcohol-content shared online [15].

Marijuana is another commonly abused substance across the nation. One study that used data from the 2013–2014 National Survey on Drug Use and Health found approximately 1 in 14 adolescents aged 12–17 have used marijuana in the past 30 days [16]. With the legalization of recreational marijuana in several states, public acceptance towards marijuana use continues to rise. In a study that examined 7000 marijuana-related tweets, sentiment analysis revealed the majority of marijuana-related tweets were pro-marijuana, with the most common themes being the intention to use or cravings for marijuana [8].

Peer influence and perceived norms also have significant influence and are highly correlated with substance abuse and risky behaviors [17–21]. The Theory of Planned Behavior posits that personal beliefs toward a behavior, perceived factors that may facilitate or hinder the performance of the behavior, and perception of social norms toward the behavior together facilitate behavioral intention [22]. In this regard, information gathered from the internet may be a means of shaping social norms for many. Therefore, we implemented a study to explore substance use mentions and indications of underage participation on Twitter.

### Study aims

The purpose of this study was to (1) describe the pattern of substance use mentions on Twitter and (2) examine popular substance items openly shared on Twitter. We created a list of keywords relating to alcohol, tobacco, substance use, and underage engagement in order to retrieve tweets about substance use and indications of underage involvement. Then, content analysis was performed on substance-related tweets to derive popular themes about substance use openly shared on Twitter, as well as patterns of underage engagement with substances.

## Methods

### Data collection

Through Twitter’s Streaming Application Programming Interface (API), we began a continuous collection of a random 1% subset of publicly available tweets with geographic coordinates. A tweet is a message of 140 or fewer characters shared on Twitter. From April 2015 to March 2016, 79,848,992 geotagged tweets across the contiguous United States were collected, of which 688,757 were identified as being related to substance use (i.e., alcohol, smoking, and recreational substance and illicit drug use). All Twitter data collected were from users who voluntarily allowed GPS coordinates to be added to their tweets. All geotagged tweets originated from the contiguous 48 states of the United States, excluding Alaska and Hawaii. The study was approved by the University of Utah Institutional Review Board. The authors have agreed and adhered to Twitter’s Terms of Service and Developer’s Agreement and Policy.

### Spatial join

We downloaded census tract boundary data from the U.S. Census Bureau’s website. The national zip code boundary data were obtained from the Environmental Systems Research Institute (ESRI). We performed a spatial join operation to associate the collected geotagged tweets with the zip codes and census tracts they fall within. Specifically, we used Python and some GIS Python libraries (Shapely, Rtree, and Fiona) to accomplish the spatial joins. R-tree was used to index the data to speed up spatial queries. After the processing, the zip code area and census tract identifiers were appended to each geotagged tweet for further statistical analysis. Some tweets (0.02%) were located in Mexico and Canada, and were thus excluded from analysis.

### Tweets processing

We processed these tweets to create indicator variables to measure substance-related mentions (e.g., alcohol, smoking, and recreational/illicit drug use) and underage substance use. We also estimated the sentiment (happy vs not happy) of each tweet. This step allowed us to further analyze whether a substance-related tweet had a positive sentiment or not. We implemented computer algorithms to process and track components of tweets, disregarding grammar and word order. Below we further describe our methods for creating sentiment scores, substance use variables, and underage variables.

#### Sentiment analysis

The first step in our process was to perform tokenization on all tweets to divide text into a sequence of tokens. A token may be words or characters such as a smiley face “:)” or other character strings. Specifically, we used PTBTokenizer to accomplish the task. PTBTokenizer can efficiently tokenizes at a rate of approximately 1 million tokens per second on a laptop computer [23]. We then matched the tokens with a list of substance keywords and underage keywords that we developed. Positive matches from this step were retrieved and included in our dataset for further processing. We implemented a machine learning Maximum Entropy Text classifier (mallet) to obtain a sentiment score for each tweet. Sentiment score ranged from 0 (sad) to 1 (happy). We classified tweets with an average mallet score equal to or greater than 0.8 as positive sentiment tweets (i.e., happy) and others as neutral/negative (i.e., sad) sentiment tweets.

#### Alcohol, smoking, and drug-related tweets

A list of 143 substance items and related phrases was developed. This list included tobacco products, alcoholic beverages, commonly abused illicit substances, common substance use phrases, and associated slang / street names compiled from the National Institute on Drug Abuse and the National Survey on Drug Use and Health [24,25]. Each item on the list was composed of one or two-words or phrases. Some items had an asterisk appended to them (“*”). An asterisk indicates the word may end with any possible spelling. For instance, “smoke* weed” captured tweets containing “smoking weed,” “smoked weed,” “smokes weed,” and “smoke weed.” Controlled substances such as marijuana and peyote were also included on this list. For each term or phrase, we tracked whether it indicated (1) alcohol (e.g., beer, vodka), (2) tobacco (e.g., cigars, e-cigarettes), or (3) drug (e.g., marijuana, cocaine) use. We did not distinguish between medical and recreational use.

To analyze substance mentions, each tweet was examined for words and phrases matching at least one substance item from the substance list. Our algorithm performed a two-step process for extracting substance use mentions. For each tweet, Step 1 was to search for matching two-word substances or phrases (e.g., magic mushroom, smoking pot). Step 2 was to search for one-word substances (e.g., LSD, cocaine) in the remaining words in the tweet.

#### Underage tweets

A list of 11 terms commonly associated with adolescents and young adults (hereafter refers as the “underage list”) was developed. For instance, "prom" is a term commonly associated with high school students. Another example is "homecoming dance," which is a dance commonly held at high schools and universities. Since the legal age for drinking and recreational marijuana use in the United States is 21, the underage list included terms associated with both adolescents and college freshmen and sophomores. While any match with items from this list does not necessarily indicate underage use, it provides a general picture of Twitter chat about substances around those under the age of 21. Specifically, the algorithm looked for any combination of words or phrases from the underage list and the substance list. For example, any match of a word or phrase from the underage list with an alcoholic beverage (e.g., beer) is considered a tweet that is both underage and drinking related, though it does not necessarily indicate underage drinking (e.g., “why do I look drunk in all my prom pictures” vs “getting drunk the night before prom”). An underage substance use tweet may indicate that the substance tweet was sent by or about someone under the age of 21.

### Quality control procedure

A quality control procedure was implemented to examine agreement rates between computer algorithm labeling and manual labeling in describing substance-related tweets. We then improved the accuracy of detecting substance-related tweets by filtering out irrelevant keywords and adding in keywords that previously had been missed.

Specifically, four random subsets of substance tweets were used to test agreement between computer and human labeling of Twitter mentions of (1) alcohol use, (2) drug use, (3) smoking, and (4) underage substance use. Additionally, we performed manual labeling for a random subset (n = 500) of all tweets collected thus far to test for inclusiveness of the substance list. Two of the co-authors manually reviewed a total of 2800 tweets: 1000 alcohol tweets, 500 drug tweets, 500 smoking tweets, 300 underage tweets, and 500 random tweets from the master dataset of all tweets. Inter-rater agreement was above 90% for all tweet subgroups. Manual labeling of these tweets yielded the following agreement rates between computer–generated labels and manually-generated labels: 88%, 86%, 75%, and 97% for alcohol, substance, smoking and underage tweets.

### Analytic approach

We grouped tweets with their associated zip code by utilizing the geo-coordinates (i.e., longitude and latitude) of where tweets were sent. We created zip code level summaries of substance use patterns according to the Twitter data. We then merged our social media dataset with the 2013 Zip Code Business Patterns from the US Census Bureau. We also merged in 2010 US Census population characteristics at the zip code level. To ease interpretation, variables were standardized to have a mean of 0 and a standard deviation of 1—with the exception of median household income, which was divided into four quartiles: income equal to or less than $47082 per year (first quartile), $47083–58401 (second quartile), $58401–73281 (third quartile), and income greater than or equal to $73281 (fourth quartile). We created indicators of income quartile to investigate whether low income and high income were both associated with increased substance use mentions. We used linear regression models to examine the associations between our Twitter-derived indicators for substance use and socioeconomic characteristics at the zip code level. Popular terms associated with alcohol, tobacco, substances, and underage tweets were identified. All statistics were performed in STATA^®^/MP 13 software.

## Results

Utilizing the substance lists as filters, a total of 687,495 substance-related tweets were detected from the master dataset of 80 million tweets. The substance dataset is heavily dominated by alcohol tweets (n = 638,347), followed by drug tweets (n = 36,284) and smoking tweets (n = 14,256). Our underage list retrieved 509 matching tweets. A match indicates that the substance tweet was tweeted by or about someone under the age of 21.

Table 1 presents the descriptive statistics of our dataset. Again, the most commonly tweeted items across all substance related tweets were alcohol-themed. When separated into detailed groups (alcohol, smoking, and drug), fewer than 10 key terms made up the majority of the tweets for each group. The top alcohol tweets included the following terms: “beer”, “drunk,” and “cocktail.” “Beer” and “drunk” alone represented 57% of all alcohol tweets (Table 2). Top smoking tweets included the terms “cigars” and “beer” (Table 2). It should be noted a tweet could have multiple substance keywords. For instance, someone could have mentioned beer and smoking hookah in one tweet. Among drug tweets, the top five terms were “get high,” “smok* weed,” “stoner,” “cocaine,” and “heroin.” The top 21 key terms that represent 90% of all substance-related tweets are presented in Table 2.

**Table 1:** Descriptive statistics of substance-related tweets.

Happiness score*	N	Mean (Standard Deviation)	Percent of tweets that are happy
All substance related tweets**	687,495	0.69 (0.20)	34.1%
Alcohol tweets	638,347	0.70 (0.20)	35.5%
Smoking tweets	14,256	0.67 (0.19)	26.4%
Drug tweets	36,284	0.56 (0.22)	12.3%
Underage engagement tweets	509	0.64 (0.23)	28.1%

* Happiness scores ranged from 0 (sad) to1 (happy). Tweets with scores ≥ 0.80 were classified as “happy.”

** Includes both controlled and recreational substances.

**Table 2:** Popular items for alcohol, smoking, and substance use tweets in descending order.

Alcohol (n = 608809)			Smoking (n = 14126)			Drug (n = 34437)			All substance-related tweets (n = 654201)		
*Terms*	*Freq*.	*%*	*Terms*	*Freq*.	*%*	*Terms*	*Freq*.	*%*	*Terms*	*Freq*.	*%*
beer	287,201	47.2%	cigars	8431	59.7%	get high	7668	22.3%	beer	317,706	48.6%
drunk	77,562	12.7%	tobacco	4386	31.1%	smok*weed	6414	18.6%	drunk	77,562	11.9%
winery	32,057	5.3%	smok* cigarette*	999	7.1%	Stoner	6259	18.2%	cocktail	44,945	6.9%
beers	30,505	5.0%	beer	310	2.2%	Cocaine	5625	16.3%	winery	32,057	4.9%
cocktail	23,343	3.8%				Heroin	2654	7.7%	tequila	21,379	3.3%
cocktails	21,602	3.6%				crack head / crackhead	1653	4.8%	alcohol	20,534	3.1%
tequila	21,379	3.5%				Shrooms	1352	3.9%	champagne	18,319	2.8%
alcohol	20,534	3.4%				got high	1139	3.3%	margarita	14,877	2.3%
champagne	18,319	3.0%				smok* pot	908	2.6%	vodka	13,228	2.0%
margarita	14,877	2.4%				on crack	765	2.2%	martini	10,836	1.7%
vodka	13,228	2.2%							margaritas	10,564	1.6%
martini	10,836	1.8%							rum	10,151	1.6%
margaritas	10,564	1.7%							booze	8,634	1.3%
rum	10,151	1.7%							cigars	8,431	1.3%
booze	8,634	1.4%							get drunk	8,017	1.2%
get drunk	8,017	1.3%							get high	7,668	1.2%
									gin	7,509	1.2%
									smok* weed	6,414	1.0%
									cocaine	5,625	0.9%
									bloody mary	5,195	0.8%
									whisky	4,550	0.7%

Keywords extracted from a total of 687495 tweets. These terms represent 90% of tweets in their respective groups. Items with an asterisk include any term stemming from that term. For example, “smok*” includes the following terms: smoked, smoker, smoking. A tweet could have more than one substance keyword.

The majority of underage tweets were about alcohol. Table 3 lists the top 19 items that accounted for 90% of underage tweets. Popular terms included “beer,” “booze,” “cocaine,” “get drunk,” “prom + weed,” and “vodka.”

**Table 3:** Most commonly tweeted substance terms among adolescents and young adults.

	*Freq*.	*%*
drunk	194	34%
beer	58	10%
alcohol	57	10%
prom + weed	26	5%
get drunk	24	4%
champagne	20	3%
winery	17	3%
vodka	16	3%
booze	13	2%
smok* weed	12	2%
got drunk	11	2%
prom + booze	11	2%
cocaine	10	2%
beers	9	2%
tequila	9	2%
brandy	8	1%
alcoholic	7	1%
cocktail	6	1%
tobacco	6	1%

These terms composed 90% of underage substance tweets. They are listed in descending order of popularity. Underage tweets (n = 509) can mention more than one substance. In total, there were 572 substance mentions among underage tweets.

Table 4 reports adjusted linear regression results in which zip code business and demographic characteristics served as predictors of percentages of tweets about 1) alcohol, 2) smoking, 3) drugs, and 4) underage engagement. More businesses in a zip code were related to lower percentages of alcohol and drug tweets. More full-service restaurants and convenience stores were associated with higher percentages of tweets about alcohol. Urbanicity was associated with fewer alcohol and drug tweets. Greater population size was associated with fewer tweets about all substances and underage engagement. A higher percentage of the zip code population who are 10–24 years of age was associated with fewer alcohol and drug tweets but more underage engagement tweets. More men in a zip code were associated with more alcohol and smoking tweets. Higher percentages of African Americans and Hispanics were associated with fewer substance-related tweets and fewer underage engagement tweets. A higher unemployment rate was associated with fewer alcohol tweets but more drug tweets. Similarly, lower median family income was associated with fewer alcohol tweets but more drug tweets (Table 4).

**Table 4:** Business and compositional predictors of alcohol, smoking, substance, and underage engagement tweets.

Zip code characteristics	% of alcohol tweets (R-squared: 0.04)	% of smoking tweets (R-squared: 0.02)	% of drug tweets (R-squared: 0.05)	% of underage tweets (R-squared: 0.44)
	Beta (95% CI)	Beta (95% CI)	Beta (95% CI)	Beta (95% CI)
*Business characteristics*				
Total number of businesses	-0.05 (-0.08, -0.02)*	0.00 (-0.04, 0.04)	-0.03 (-0.06, 0.00)*	-0.04 (-0.17, 0.08)
Alcohol places	0.01 (0.00, 0.03)*	0.00 (-0.02, 0.01)	0.00 (-0.02, 0.01)	0.00 (-0.04, 0.03)
Full service restaurants	0.03 (0.01, 0.06)*	0.01 (-0.02, 0.05)	-0.02 (-0.05, 0.01)	0.00 (-0.09, 0.09)
Fast food restaurants	0.02 (-0.02, 0.05)	-0.02 (-0.07, 0.03)	0.03 (0.00, 0.07)	0.05 (-0.07, 0.18)
Grocery stores and supermarkets	0.01 (-0.01, 0.03)	0.00 (-0.02, 0.03)	0.00 (-0.01, 0.02)	0.01 (-0.08, 0.09)
Convenience stores	0.06 (0.05, 0.08)*	0.00 (-0.02, 0.02)	-0.01 (-0.02, 0.01)	-0.01 (-0.06, 0.04)
*Demographic and economic characteristics*				
Urban (yes/no)	-0.11 (-0.15, -0.07)*	0.02 (-0.08, 0.12)	-0.22 (-0.27, -0.16)*	-0.17 (-0.41, 0.07)
Population size	-0.11 (-0.13, -0.09)*	-0.06 (-0.10, -0.02)*	-0.08 (-0.11, -0.05)*	-0.17 (-0.28, -0.06)*
Population density	0.01 (-0.01, 0.02)	0.01 (-0.01, 0.04)	0.01 (0.00, 0.03)	0.04 (-0.03, 0.11)
Percent 65 years+	0.02 (-0.01, 0.04)	0.03 (-0.02, 0.09)	0.01 (-0.03, 0.04)	0.03 (-0.13, 0.19)
Percent 10–24 years	-0.06 (-0.08, -0.03)*	0.02 (-0.02, 0.07)	-0.06 (-0.09, -0.03)*	0.14 (0.06, 0.23)*
Percent male	0.07 (0.04, 0.09)*	0.13 (0.08, 0.19)*	0.01 (-0.03, 0.05)	0.02 (-0.17, 0.21)
Percent African American	-0.05 (-0.06, -0.03)*	0.01 (-0.03, 0.05)	-0.06 (-0.08, -0.04)*	-0.17 (-0.27, -0.08)*
Percent Hispanic	-0.01 (-0.03, 0.01)	-0.05 (-0.09, -0.01)*	-0.06 (-0.08, -0.03)*	-0.17 (-0.26, -0.08)*
Household size	-0.03 (-0.06, 0.00)	0.13 (0.08, 0.19)*	0.06 (0.03, 0.10)*	0.08 (-0.06, 0.21)
Unemployment rate	-0.05 (-0.08, -0.02)*	-0.06 (-0.14, 0.02)	0.08 (0.03, 0.12)*	-0.16 (-0.34, 0.02)
Percent less than high school graduate	0.01 (-0.02, 0.03)	-0.07 (-0.15, 0.00)	-0.03 (-0.07, 0.02)	1.01 (0.85, 1.16)*
Median family income, 4th quartile (highest)	0.08 (0.02, 0.13)*	-0.11 (-0.25, 0.03)	-0.20 (-0.28, -0.12)*	-0.02 (-0.33, 0.28)
Median family income, 3rd quartile	0.02 (-0.03, 0.07)	-0.07 (-0.20, 0.05)	-0.15 (-0.22, -0.07)*	-0.03 (-0.30, 0.24)
Median family income, 2nd quartile	0.00 (-0.05, 0.04)	0.07 (-0.05, 0.18)	-0.10 (-0.17, -0.03)*	-0.04 (-0.31, 0.23)
Sample size	17377	3924	9151	425

All variables are standardized to have a mean of 0 and standard deviation of 1.

*p<0.05.

The distribution of substance-related tweets is shown in Table 5. Each entry denotes the proportion of tweets for the column (alcohol, smoking, drug) in a month. For instance, 20.1% of all alcohol-related tweets were captured in April. Of all alcohol, smoking, and drug-related tweets captured between April 2015 and March 2016, the largest proportion of tweets occurred in April. The second largest proportion of smoking-related content occurred in July, while May captured the second highest record for alcohol and drug-related tweets. March had the fewest alcohol, smoking, and drug-related tweets, and the frequency (not presented) of mentions was at its lowest in the 12-months period.

**Table 5:** Distribution of substance-related tweets, by month.

	*Alcohol*	*Smoking*	*Drug*
January	5.1%	5.7%	2.1%
February	6.2%	7.0%	2.0%
March	2.7%	3.2%	0.8%
April	20.1%	18.5%	56.7%
May	11.6%	9.8%	10.5%
June	6.0%	5.7%	4.6%
July	10.9%	10.0%	6.3%
August	9.0%	9.0%	4.9%
September	6.5%	6.5%	3.3%
October	8.2%	8.5%	3.2%
November	7.2%	8.5%	3.0%
December	6.7%	7.7%	2.6%

Tweets were collected from the continental United States from April 2015 to March 2016. Each entry denotes the percent of tweets for the column (alcohol, smoking, drug) in a month. For instance, 20.1% of all alcohol-related tweets were captured in April.

**Fig 1:**
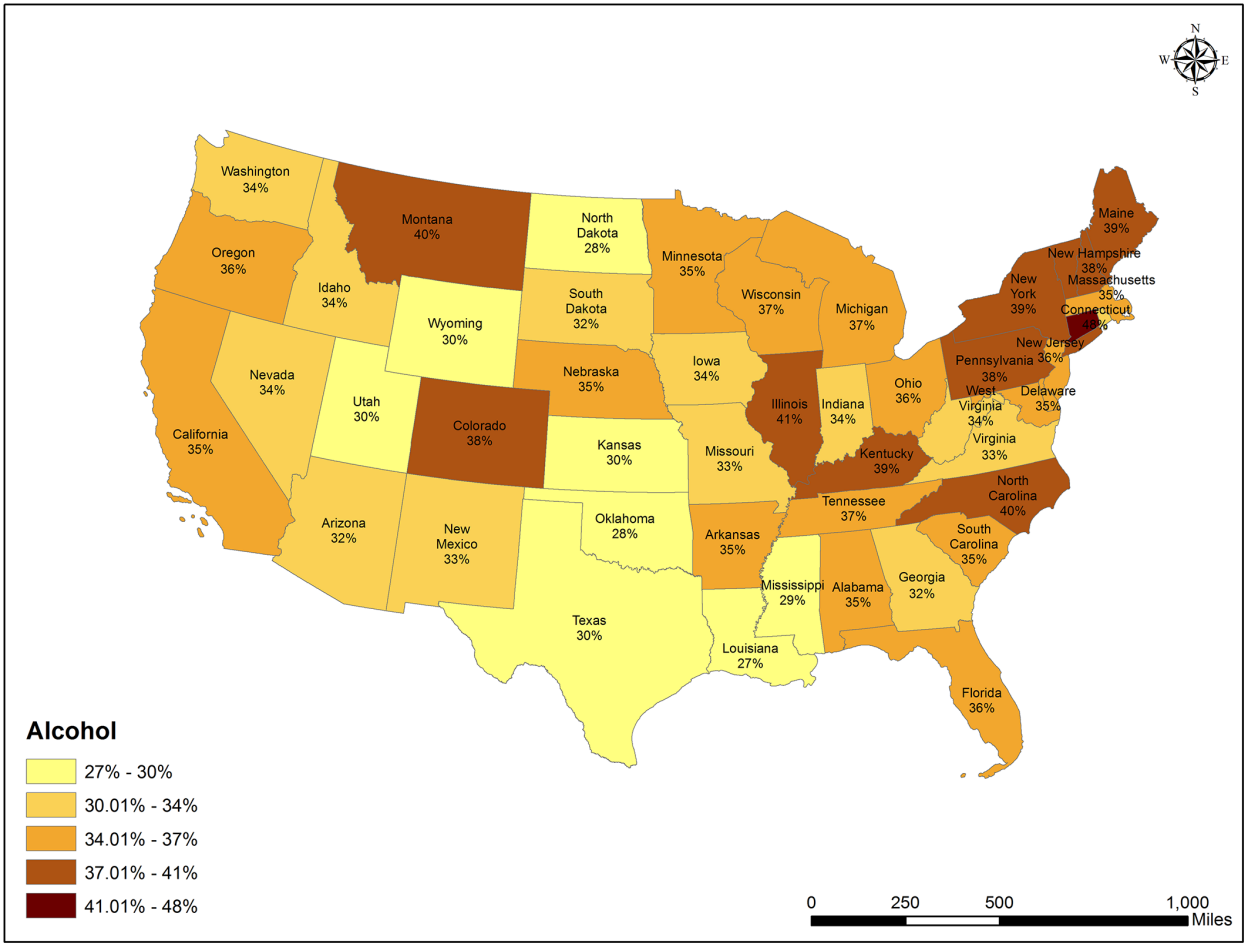
Percent of alcohol-related tweets that are happy, by state.

S1 Table shows the percent of substance-related tweets that were “happy” in each state. When tweeting about alcohol, smoking, and drug-related content, the following states had the largest percentages of happy tweets for each category, respectively: Connecticut (48%), Nebraska (54%), and South Dakota (35%). Overall, the percent of happy tweets ranged from 27% to 48% for alcohol, 9% to 54% for smoking, and 7% to 35% for drug use. Additionally, the northeastern region of the United States had higher percentages of happy tweets relating to alcohol (Fig 1). Maps displaying the national distribution of smoking and drug-related happy tweets are presented in S1 and S2 Figs.

## Discussion

In this study, we examined patterns in publically shared substance mentions on Twitter by analyzing a 1% random sample of tweets collected over a 1 year period at the national level. Across all substance tweets, alcohol was the most commonly tweeted theme. The most widely used terms included “beer,” followed by “drunk” and “cocktail,” which is consistent with the findings by Cavazos-Rehg and colleagues [14]. Additionally, a significant portion of smoking tweets also mentioned beer (Table 2). This finding indicates a large proportion of individuals tweeted about smoking and alcohol simultaneously. In our study, we observed a high percentage of substance-related tweets were happy (34%), which is higher than the previously reported percentages of happy tweets about food and physical activity [26].

Nearly 9 of every 1000 tweets were alcohol-related. This finding is slightly higher than a previous finding in 2014 that 1 in every 1250 tweets were drinking-related [14]. One plausible explanation is that social media use and smartphone use (which enables geotagging of tweets) have increased steadily over the years. For instance, reports from Pew Research Center suggest smartphone access for teens ages 12–17 have increased from 37% in 2012 to 73% in 2015 [27,28].

Most of the underage substance use tweets from this study were alcohol-themed, followed by marijuana use. With the legalization of recreational use of marijuana, marijuana tweets unsurprisingly ranked the highest in drug-related tweets (Table 2). However, legalization of recreational use increases ease of access, which can have negative impacts on adolescents and increase drug dependency and abuse. Online discussions also have the potential to glorify and promote substance use [14,29]. For instance, some individuals may be more interested to engage in risky activities after seeing such activities being promoted by friends or people they follow. Through Twitter features such as retweet (i.e., reposting the tweet) and favorite (i.e., liking a tweet), the reach of a tweet can be widespread.

Our data also showed substance-related tweets were associated with business and demographic characteristics of zip codes. For instance, areas with higher concentration of convenience stores were associated with higher percentages of tweets about alcohol. This suggests a potential increased use of alcohol for people who reside or spend more time in an area with higher density of convenience stores. This is demonstrated in one study where increased risk of underage drinking was related to the availability of convenience stores near a school [30]. However, in another study that examined alcohol access with diverse adolescent population, commercial sources including convenience stores were not primary sources for youths to access alcohol [31]. Furthermore, our finding regarding convenience stores differs from a study with African American drinkers. In that study, convenience stores density was not associated with increased risk of alcohol consumption [32]. Such mixed literature findings may be attributed to differences in convenience store shoppers’ preferences and cultural habits across various subpopulations and locations.

Additionally, greater population size was significantly associated with fewer mentions about substance use and underage engagement. On alcohol and drug use, our finding is somewhat consistent with an earlier study by Parker, Weaver, and Caihoun [33] wherein residence in a non-metropolitan area influenced alcohol consumption for Hispanic participants but not for the White participants. The same study also found residence in an area with population exceeding 1 million was a significant predictor for drug use among the White participants. Another study with a national sample found more reports of nonmedical use of prescription opioids among residents of rural counties than their counterparts in urban counties [34]. Regarding underage engagement with substance use, our finding aligns with several previous studies wherein adolescent substance use including nonmedical use of prescription drugs was more prevalent in rural than urban areas [35,36]. For instance, one study found 8th graders who lived in rural areas were 83% more likely to have used cocaine than their peers who lived in urban areas [35]. Greggo, Jones, and Kann [37] also found high school students in rural areas were more likely to have reported driving after drinking than students in urban areas. One possible explanation for higher prevalence of risky behaviors in areas with lower population density may be that social norms may spread easier in environments with higher social cohesion. Permissive social norms may also contribute to engagement of undesirable health behavior in areas with higher level of collective efficacy [38]. Finally, residents in rural areas may be more susceptible to social stigma which contributes to less utilization of mental health services [39]. Although literature is mixed on whether smaller population size leads to greater or less substance use, studies have demonstrated substance use can be observed anywhere regardless of an area’s urbanicity or population size [35,37,40]. People in smaller towns can engage in risky behaviors, or have discussions about substances and illicit drug uses online, just as others can in bigger cities.

Our data also showed areas with higher percentages of African Americans and Hispanics were associated with fewer mentions about substance use and underage engagement. This finding aligns with the results of one study where African and Hispanic American high school students had lower rates of binge drinking [41]. One plausible explanation is that traditional cultural norms with protective factors such as strong family ties and conservative cultural values may act as a buffer to lessen engagements with substance use. Previous research has suggested that ethnic identity may be a protective factor against substance use and risky behaviors [42,43].

It is also notable that economic disadvantage was associated with fewer alcohol tweets but more drug tweets. Previous research has identified a relationship between neighborhood disadvantage and increased drug use, that is partially mediated by social stressors and higher psychological distress among residents [44]. Alternatively, the literature on neighborhood disadvantage and alcohol use is mixed, with some studies finding null relationships [45,46] and other studies finding increased alcohol consumption with neighborhood disadvantage [47,48]. The pattern of drinking may vary by socioeconomic status. People of higher socioeconomic status and those living in more affluent areas may be more likely to use alcohol than their lower socioeconomic status counterparts [49]. Heavy episodic drinking may be more prevalent in economically disadvantaged areas [47]. However, effects of neighborhood characteristics may be modified by racial/ethnic status and gender of individuals. A previous study found neighborhood disadvantage was related to more negative consequences of drinking with indirect pathways through increased heavy drinking and pro-drinking attitudes, but only among racial/ethnic minority men. Also among minority men, immigrant composition of neighborhoods was related to fewer alcohol problems. Neighborhood disadvantage was not related to alcohol consumption or alcohol problems among white men, white women or minority women [50].

### Strengths and limitations

In this study, we leveraged social media data to examine current trends and patterns of substance use. Our results contribute to a growing literature utilizing social media as well as machine learning methods to examine health topics and monitor trends [51–55]. Our national data collection spanned one year which allowed us to capture tweets that were holiday-related and took into account different time periods involving high and low tweet traffic (e.g., summer break, Thanksgiving). The one year collection allowed us to capture and analyze over 688,000 substance related tweets through collaboration between fields of health promotion, computer science, and geography. Promising agreement rates between our algorithm-labeled and manual-labeled tweets also strengthen our findings. Additionally, the tweets we examined are publicly available and thus, can influence a wide audience. Furthermore, social modeling via social media may be an important emerging way in which adolescents and young adults obtain information about substances and social media may help to normalize substance use behaviors.

This study is subject to several limitations. All data collected thus far are from a 1% sample of publically available Twitter data. Our substance list only captured a small sample of tobacco tweets. While it may be that users simply tweeted more about alcohol and other substances, this may also indicate a need to further refine the substance list. Similarly, the underrepresentation of underage tweets may be due to a need to further expand the list of keywords. It should be noted that the geo-coordinates associated with a tweet pertain to where the tweet was sent rather than the location of a person’s residence. Thus, summaries of the social environment for a given area utilized tweets sent by individuals who were present at the location, and can be a mixture of visitors and residents. However, because tweets analyzed composed a random sample, the algorithm is naturally weighted towards people who spend more time in an area. Additionally, the underage list was created with the intention to capture tweets with words or phrases associated with underage substance use. However, the list did include terms that may apply to those over the age of 21 (i.e., college juniors and seniors as well as graduate students may also attend “homecoming dance”). Finally, we cannot conclude that a high frequency of substance use mentions necessarily indicates a high use of substances or confirm the intention to use them.

### Study implications

The amount of publically sharing of alcohol and substance-related messages is alarmingly high. Moreover, we find that substance-related tweets occurred more in areas with smaller population sizes and fewer racial/ethnic minorities. Additionally, publically available tweets have the potential to normalize illicit drug use and risky behaviors as tweets reach a wide range of audiences [14,29]. On top of the potential to endorse risky behaviors such as underage drinking and drug abuse, Twitter and other social networking sites may have the potential to encourage initiation into substance use. Substance-related discussions may act like an invitation for individuals to engage in risky behaviors, especially adolescents who are particularly influenced by friendship norms. Social influence is central to substance abuse and underage drinking. Health educators and health promotion specialists may take the rise of social media as an opportunity to develop interactive online campaigns to prevent the potential for normalization of risky behaviors.

## Supporting information

S1 Fig Percent of smoking-related tweets that are happy, by state. (TIF)

S2 Fig Percent of drug-related tweets that are happy, by state. (TIF)

S1 Table Percent of substance-related tweets that are happy, by state. (PDF)
