# Supplementary figures and images for: National substance use patterns on Twitter
Source: PLoS One. 2017 Nov 6;12(11):e0187691. doi: 10.1371/journal.pone.0187691 (PMC5673183; doi:10.1371/journal.pone.0187691)

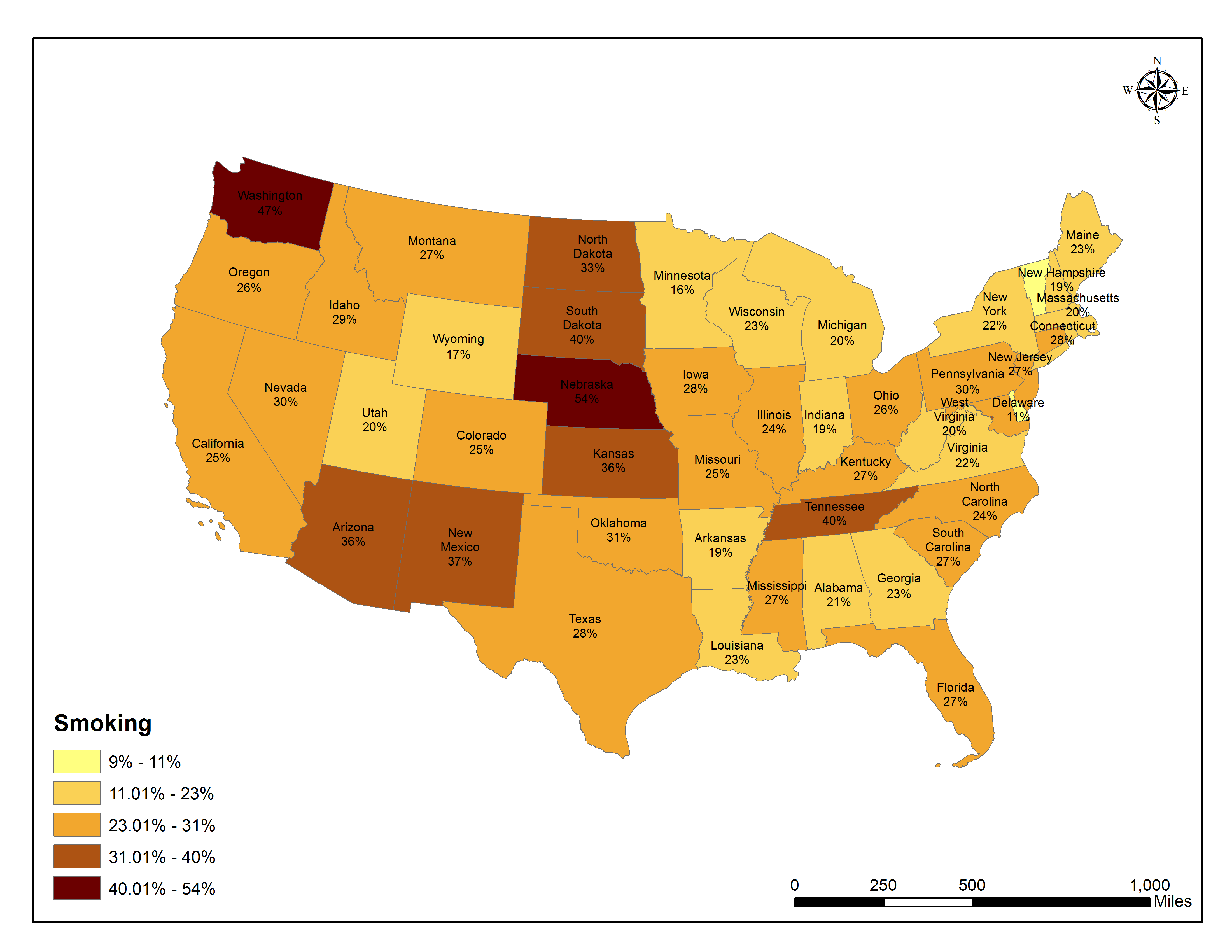

Supplement: S1 Fig — (TIF) [file pone.0187691.s001.tif]

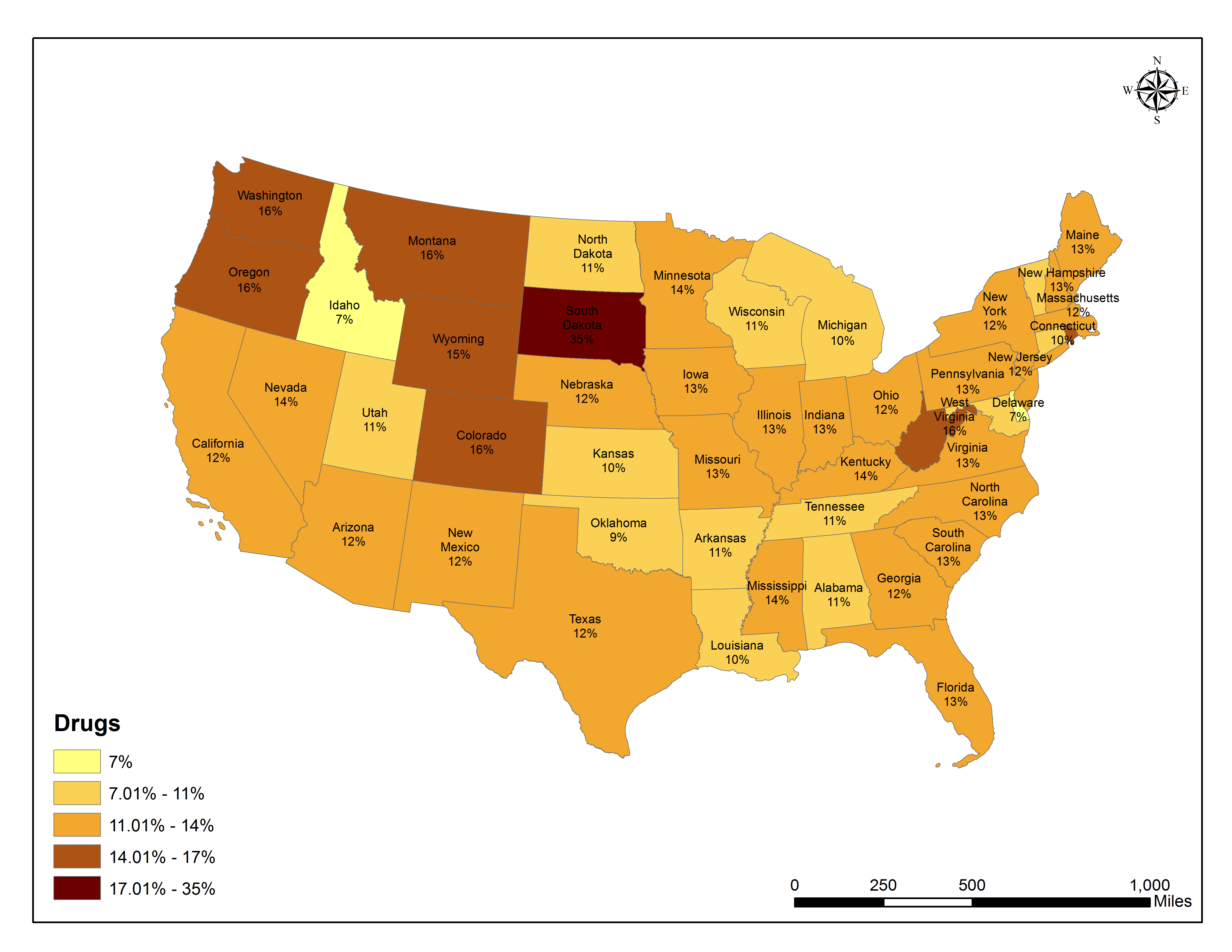

Supplement: S2 Fig — (TIF) [file pone.0187691.s002.tif]
