# Supplementary material for: National substance use patterns on Twitter
Source: PLoS One. 2017 Nov 6;12(11):e0187691. doi: 10.1371/journal.pone.0187691 (PMC5673183; doi:10.1371/journal.pone.0187691)
Supplement: S1 Table — (PDF) [file pone.0187691.s003.pdf]

S1 Table. Percent of substance-related tweets that are happy, by state

State	*Alcohol*	*Smoking*	*Drug*
Alabama	35%	21%	11%
Arizona	32%	36%	12%
Arkansas	35%	19%	11%
California	35%	25%	12%
Colorado	38%	25%	16%
Connecticut	48%	28%	10%
Delaware	35%	11%	7%
District of Columbia	36%	32%	11%
Florida	36%	27%	13%
Georgia	32%	23%	12%
Idaho	34%	29%	7%
Illinois	41%	24%	13%
Indiana	34%	19%	13%
Iowa	34%	28%	13%
Kansas	30%	36%	10%
Kentucky	39%	27%	14%
Louisiana	27%	23%	10%
Maine	39%	23%	13%
Maryland	36%	25%	11%
Massachusetts	35%	20%	12%
Michigan	37%	20%	10%
Minnesota	35%	16%	14%
Mississippi	29%	27%	14%
Missouri	33%	25%	13%
Montana	40%	27%	16%
Nebraska	35%	54%	12%
Nevada	34%	30%	14%
New Hampshire	38%	19%	13%
New Jersey	36%	27%	12%
New Mexico	33%	37%	12%
New York	39%	22%	12%
North Carolina	40%	24%	13%
North Dakota	28%	33%	11%
Ohio	36%	26%	12%
Oklahoma	28%	31%	9%
Oregon	36%	26%	16%
Pennsylvania	38%	30%	13%

Rhode Island	34%	21%	17%
South Carolina	35%	27%	13%
South Dakota	32%	40%	35%
Tennessee	37%	40%	11%
Texas	30%	28%	12%
Utah	30%	20%	11%
Vermont	40%	9%	11%
Virginia	33%	22%	13%
Washington	34%	47%	16%
West Virginia	34%	20%	16%
Wisconsin	37%	23%	11%
Wyoming	30%	17%	15%
